# Pancreatic Dysfunction Masquerading as an Insulinoma

**DOI:** 10.7759/cureus.38697

**Published:** 2023-05-08

**Authors:** Siddharth Shah, Christian Scott, ShahZeib Syed, Safia Afaq, Priscilla Fujikawa

**Affiliations:** 1 Internal Medicine, LewisGale Medical Center, Salem, USA

**Keywords:** clinical case report, case-report, pancreatic neuroendocrine tumors, pancreatic insulinoma, pancreatic insufficiency

## Abstract

Insulinoma is a rare neuroendocrine tumor that overproduces insulin, resulting in hypoglycemic symptoms. Elevated C-peptide levels in the absence of sulfonylurea use indicate insulinoma. Treatment is usually glucose administration and if the tumor size is large, surgery may be warranted. We present a case of a young man who had a one-year continuing episode of hypoglycemic symptoms that resolve after consuming high-glucose solids and liquids. Although symptoms pointed toward insulinoma, the 72-hour fasting test failed to show insulinoma. This case shows how following the algorithm accurately will prevent an inaccurate diagnosis.

## Introduction

Insulinoma is a rare neuroendocrine tumor that overproduces insulin and is prevalent in one to four million people [1]. During insulin-induced hypoglycemia, symptoms have been seen in glucose levels of 60 mg per deciliter [2]. Symptoms of hypoglycemia result due to autonomic nervous system or lack of glucose going to the brain [2]. Symptoms seen include diaphoresis, tremors, palpitations, confusion, and visual disturbances [2]. Diagnosis is often by 72-hour supervised fast performed in a hospital under the standardized protocol. Imaging is used to localize tumors if surgery is indicated. Most patients if operated on are cured by surgery; however, many can be managed medically as well. Medical therapy includes drugs like diazoxide, verapamil, phenytoin, propranolol, and octreotide [2]. In our case, it is shown how the supervised fasting test prevented an inaccurate diagnosis.

## Case presentation

A 27-year-old Caucasian male with no past medical history initially presented to his primary care physician’s office with symptoms of dizziness, nervousness, tremors, and fatigue for one month. The patient reported symptoms worsening with fasting for more than 24 hours and relieved with consuming high glucose foods and liquids. He is a non-smoker, a non-drinker has no recreational drug use, nor has he used any steroids in the past. Workup at his primary care physician revealed high insulin levels; therefore, he was scheduled for a routine MRI on an outpatient basis. MRI revealed a focal 12mm by 8mm enhancement in the posterior tail of the pancreas. After the MRI results, the primary care physician referred the patient to a general surgeon who admitted the patient to the hospital. The time frame from symptom onset to hospital admission was two months.

Upon admission, the patient was afebrile, blood pressure 150/92 mmHg, pulse 86 bpm, and respiratory rate was 16. On physical examination, he was not in any acute distress, abdomen was soft, obese, and nontender. No hepatosplenomegaly or masses were noted. CT scan of the abdomen done in the hospital did not show any pancreatic enhancement. Results of a comprehensive metabolic panel before the 72 hours fast is shown in Table 1.

**Table 1:** Comprehensive metabolic panel before 72-hour fast

Laboratory Markers	Laboratory Values	Reference Range
Glucose	78 mg/dL	90-130 mg/dL
Beta-Hydroxybutyrate	0.18 mmol/L	0.02-0.27 mmol/L
C-Peptide	3.0 ng/mL	0.78-3.9 ng/mL
Insulin	20.1 ulU/mL	2-20 uIU/mL
Proinsulin	7.2 pmol/L	0-8 pmol/L

Shortly after admission, a 72-hour fast was started with glucose checks every two hours per endocrinology recommendations. Glucose levels stayed in 60s and 70s with lowest being 59 mg/dL. The patient ended up not qualifying for diagnosis of insulinoma and likely had pancreatic dysfunction of unknown. The patient discharged with outpatient follow up with General Surgery and Endocrinology.

During the general surgery appointment, patient was not worked up nor did he have any symptoms at that point, therefore, no follow up visit was scheduled. During endocrinology follow up, metanephrines, serotonin, and thyroid stimulating hormone levels were normal. Patient was advised to check glucose daily. If a recurrence of symptoms were to occur, the plan was to do mixed meal test. Follow-up visit was scheduled one month after; however, patient canceled the appointment as he no longer had symptoms.

## Discussion

The association of hyperinsulinism and a functional islet cell tumor was initially described by Wilder et al. in 1927 with the first surgical cure being achieved by 1929 [1]. Whipple made an observation later in time describing symptoms of hypoglycemia in the setting of fasting, circulating glucose of <50 when symptomatic, and relief of symptoms with the administration of glucose [1]. This constellation of symptomatology was later referred to as Whipple's Triad [1]. A diagnostic test for insulinoma is called the 72-hour supervised fasting test. A positive test would show increased levels of C-peptide and insulin in the presence of hypoglycemic symptoms [2]. Imaging is often used to localize the tumor for surgical planning to anatomically localize intraoperatively [2]. Insulinoma is often benign and may be seen with multiple endocrine neoplasia type 1 (MEN1) syndrome [2]. The treatment of choice for insulinoma is surgical excision which cures the majority of patients [3]. For patients who are not candidates for surgical resection, medical treatments such as diazoxide and octreotide are used [3].

MEN1 syndrome includes parathyroid and pituitary glands as well as pancreatic involvement. Our patient had a negative CT scan of the abdomen and a negative 72-hour supervised fasting test excluding insulinoma, and thus MEN1 syndrome. Our patient denied any use of sulfonylurea use which was supported by her C-peptide value in Table 1.

There are many causes of hypoglycemia, which include excessive alcohol drinking which can keep the liver from releasing glucose from its glycogen stores into the bloodstream. Severe hepatitis, cirrhosis, advanced heart disease, long-term starvation, and adrenal gland tumors can also cause hypoglycemia [3].

## Conclusions

Insulinoma is a very rare tumor. Although this patient had many hypoglycemic symptoms and imaging pointing towards insulinoma, 72 hour supervised fasting test was negative. Repeat CT scan in hospital was negative for any lesions or nodule. Patient improved after discharge and did not need any further workup. This report shows how following the algorithm to accurately diagnose prevented misdiagnosis.
